# Alternative medicines for AIDS in resource-poor settings: Insights from exploratory anthropological studies in Asia and Africa

**DOI:** 10.1186/1746-4269-4-16

**Published:** 2008-07-10

**Authors:** Anita Hardon, Alice Desclaux, Marc Egrot, Emmanuelle Simon, Evelyne Micollier, Margaret Kyakuwa

**Affiliations:** 1 Amsterdam School for Social Science Research, University of Amsterdam, The Netherlands; 2 Centre de Recherche Cultures, Santé, Sociétés (CReCSS), Université Paul Cézanne d'Aix-Marseille, Aix-en-Provence, France; 3 Institut de Recherche pour le Développement (IRD), Unité Mixte de Recherche (UMR) 145, Montpellier, France; 4 UMR 7043 Centre National de Recherche Scientifique (CNRS)-Université Marc Bloch, Strasbourg, France

## Abstract

The emergence of alternative medicines for AIDS in Asia and Africa was discussed at a satellite symposium and the parallel session on alternative and traditional treatments of the AIDSImpact meeting, held in Marseille, in July 2007. These medicines are heterogeneous, both in their presentation and in their geographic and cultural origin. The sessions focused on the role of these medications in selected resource poor settings in Africa and Asia now that access to anti-retroviral therapy is increasing. The aims of the sessions were to (1) identify the actors involved in the diffusion of these alternative medicines for HIV/AIDS, (2) explore uses and forms, and the way these medicines are given legitimacy, (3) reflect on underlying processes of globalisation and cultural differentiation, and (4) define priority questions for future research in this area. This article presents the insights generated at the meeting, illustrated with some findings from the case studies (Uganda, Senegal, Benin, Burkina Faso, China and Indonesia) that were presented. These case studies reveal the wide range of actors who are involved in the marketing and supply of alternative medicines. Regulatory mechanisms are weak. The efficacy claims of alternative medicines often reinforce a biomedical paradigm for HIV/AIDS, and fit with a healthy living ideology promoted by AIDS care programs and support groups. The AIDSImpact session concluded that more interdisciplinary research is needed on the experience of people living with HIV/AIDS with these alternative medicines, and on the ways in which these products interact (or not) with anti-retroviral therapy at pharmacological as well as psychosocial levels.

## Alternative medicines for AIDS in resource-poor settings: insights from exploratory anthropological studies in Asia and Africa

A large number of new treatments offered to people living with HIV/AIDS (PLWA) have appeared over the last fifteen years in the therapeutic domain of AIDS. These medicines are particularly heterogeneous, both in their presentation and in their geographic and cultural origin. They constitute a group of products with a therapeutic aim that occupies a space between the customary traditional, popular and biomedical sectors of health care [1]. These products often mix reference to biomedicine and science with notions of traditional health culture and nature in a syncretic way. They consist mainly of herbs and nutritional substances and are packaged as 'modern' pharmaceuticals: capsules, tablets, and solutions. The names of these alternative treatments reflect their reference to biomedicine: Immunocomplex, Viralgic, Virjint, etc. Their accompanying leaflets provide detailed information on substance, as well as dosage, indications, and biomedical efficacy claims. Their diffusion follows contemporary paths in the global economy and makes use of new information technologies. In this paper, we will use the term "alternative" to consider a generic category including medicines that recently appeared for AIDS which have not been authorised by drug regulatory authorities, nor recommended by WHO. Other terms, such as neo-traditional or neo-phytotherapeutic, may be discussed for the characterization of some of these treatments, related to their local meanings or their social status.

The emergence of alternative medicines for AIDS in Asia and Africa was discussed at a satellite symposium and the parallel session on alternative and traditional treatments of the AIDSImpact meeting, held in Marseille, in July 2007. We were especially interested in the role of these medications since the introduction and rapid scale-up of highly active anti-retroviral therapy (HAART) in resource poor settings.

Twenty anthropologists and health researchers attended the satellite session and presented exploratory findings from Asia and Africa (Uganda, Senegal, Benin, Burkina Faso, China and Indonesia). The aims of the satellite, the results of which were presented at the parallel session [2], were to (1) identify the actors involved in the diffusion of these alternative medicines for HIV/AIDS, (2) explore uses and forms of these medicines, and the way they are given legitimacy, (3) reflect on underlying processes of globalisation and cultural differentiation, and (4) define priority questions for future research in this area. We present here the insights generated at the meeting, illustrated with some findings from the studies that were discussed.

## Modernization of traditional medicine

There has been an increased professionalisation and commercialisation of traditional medicine in response to the development of biomedicine. This trend is not specific to AIDS and not necessarily a recent development. Social scientists first noted this trend in the late 1980s: Charles Leslie [3] for example has shown how, in India, in response to an increased authority of biomedicine and the globalisation of health markets, Unani and Ayurvedic medicine production changed; and Afdhal and Welsch [4] described the rise of 'modern' *jamu *in Indonesia. *Jamu *is the traditional term for Indonesian indigenous medicines usually prepared from herbal medicines such as leaves, bark, roots and flowers. Nowadays a multimillion dollar industry is involved in the production of Ayurvedic and Unani medicines in India, and of *jamu *in Indonesia. A rapidly expanding assortment of powders, creams, pills, capsules and cosmetics has been manufactured both in small cottage industries as in large factories with increasingly sophisticated technologies [3,4]. The modernization of the manufacturing of these drugs has been accompanied with more modern biomedical modes of presenting their efficacy [5]. Under globalization, similar trends occurred in other regions and these products diffused more rapidly.

At the seminars in Marseille, we discussed the ways in which such alternative remedies operate in the therapeutic domain of AIDS care. In the first decade of the AIDS epidemic there was no effective treatment for HIV/AIDS and patients were faced with nearly certain premature death. At that time, there were regular hypes offering hope for life. But with the introduction of ART, alternative treatments are now marketed for many additional purposes too: to prevent AIDS, to kill viruses, to delay the need for ART, to restore and enhance health while on ART, to treat opportunistic infections, and to alleviate adverse side effects of other treatments. Biomedical practitioners generally discourage the use of alternative medicines, fearing interactions with ART and also through the concern that patients may stop using ART.

## Supplying alternative medicines for HIV/AIDS

**Figure 1:**
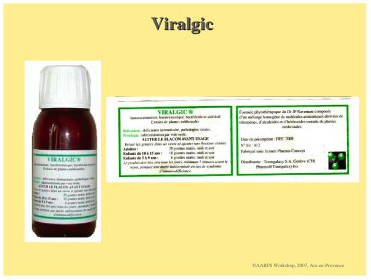
Viralgic, West Africa.

At the AIDSImpact sessions Egrot and colleagues [6] presented findings on the supply of what they label "neo-traditional medicines" to refer to the boundary-crossing nature of these treatments in West Africa. The "designers" of the inventoried products are extremely heterogeneous. In some cases these people are nationals of African countries who present themselves as healers. Some say they have undertaken "research" on the basis of therapeutic products that were already known locally. Others refer to a dream revelation (classic in the universe of healers in Africa) of a plant composition that is "efficacious" against AIDS, while yet others speak of a divine revelation. Physicians, scientists and academics are solicitated, brought into involvement or spontaneously engage themselves in the exploitation of neo-traditional products. The case studies in West Africa show that other treatments, such as Immunicomplex or Aloe Vera, originate in Europe and the USA. Alternative medicines from Europe and the USA occupy the same shelves in ordinary pharmacies as those originating from Africa and China, often along with a few 'immune-boosting' food products (honey, olive oil). Specialized "bio", "natural health" and "health food" shops make these products available to the more affluent. The distributors and marketing men of these products also target health workers and clinics directly. The West Africa case studies noted that health workers also have started to prescribe alternative products such as Immuboost (NHi2T) or Viralgic (Pharma Concept) (see Figure 1).

A case study from Uganda showed how health workers operating an anti-retroviral treatment program adopted a locally available traditional ointment as an alternative medication for skins problems of people living with HIV and AIDS. The skin problems result from adverse effects of ART or symptoms of opportunistic infections. The health workers obtained the recipe from local traditional healers (patients had told them that the cream works well), and the patients help collect the ingredients. They 'repackage' this traditional remedy into what is now called 'mobile cream' (to make clear it is produced by the so called 'Mobile' ART program). One of the nurses reports:

"*The mobile cream, which we ourselves prepare either at our chief nurse's home or here at the office depending on how busy we are at the office, is very efficacious for many kinds of skin related conditions. We are quick to prescribe it to the patients because we know it works and it is popular among patients too because it works for them*[7]."

**Figure 2:**
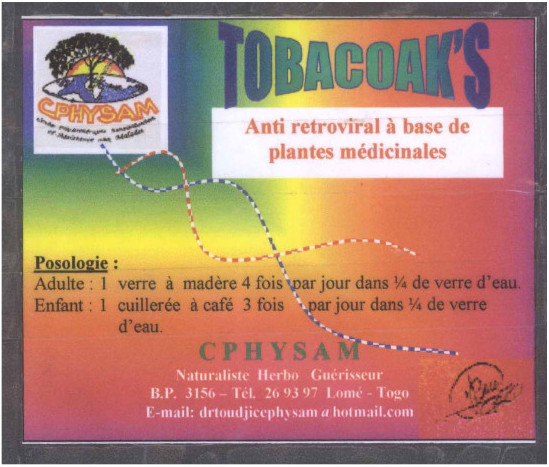
Tobacoak's, West Africa.

Content analysis of drug information leaflets, advertisements, product catalogues, and brochures distributed by medical representatives in the West Africa case studies [6,8] casts light on the range of effects that are attributed to these drugs. Most commonly cited (biomedical) properties are immune-stimulation and antioxidant. Some manufacturers suggest that the products have antiviral properties as well. The antiviral dimension refers either to the opportunistic infections such as herpes (mentioned for example in the product information for Immuboost) or eventually to the immunodeficiency syndrome itself. Indeed, some products boldly claim anti-HIV activity as well, and are marketed as natural ART (see Figure 2).

However, such efficacy claims are not static. The producer of Virusinest (Nesto-Pharma) recently withdrew the antiviral claim, stating in its information leaflet: "the analyses carried out among patients do not allow the anti-HIV assertion to be upheld". There may be also inconsistencies between various information sources. The brochure for Viralgic (Pharma Concept) says that this is a product which renders the virus undetectable, but the website of the manufacturer presents the drug as immunostimulant (result of trials published on the web site), and present the product as treatment for opportunistic infections: "anti-herpes...for healthy persons".

## The demand for alternative medicines

A case study on Indonesia [9] dealt with the demand for alternative medicines among PLWA. As Afdhal and Welsch noted two decades ago, Indonesia has a thriving market for *jamu*. *Jamu *are sold for a wide range of indications: common colds, influenza, headaches, aches and pains, high blood pressure, beauty, improvements in sexual performance, and recently to treat and prevent HIV-related health problems. AIDS prevalence is below 1% (i.e. this is a low prevalence area), but the disease is stigmatised, because of its association with intravenous drug use and prostitution. Hardon and her colleagues conducted interviews with women and men who live with HIV and use anti-retroviral therapy, mainly intravenous drug users and their partners. All of them had better health since taking these modern drugs. Nonetheless, all of them see the need to take *jamu *as well. They do so in part out of their intention to live positively (i.e. eating and sleeping well, and keeping a positive outlook on life), as promoted by many of the support groups in which PLWA participate. The respondents do not make distinctions between modern medicines and *jamu *in these health maintenance and restoring practices. Rather they distinguish the drugs by their effects. They use popular *jamu *to treat side effects of HAART, such as itchiness. These *jamus *are not specifically promoted for HIV and AIDS in Indonesia, perhaps partly because the disease is so stigmatised.

However one neo-traditional preparation stood out in the narratives of our respondents as a product which can treat HIV/AIDS: virgin coconut oil. Ceri, for example, started using coconut oil shortly after she found out she was HIV-positive. She says:

*Actually, the effect is not only for your immune system. So, I feel better, don't feel tired, and have more energy. I think what influences most is self-suggestion. It's self-suggestion that matters*...

She stopped taking the oil because it is very expensive.

Mia (a 28 year old woman from Jakarta) was given virgin coconut oil by a friend from Yogjakarta:

*I got 70 boxes. A box contains 60 capsules. It took it every day until I felt sick, but there was no effect. My CD4 level did not increase. Three months, three months made me look like a coconut you just needed to squeeze (laughing). I became very oily. The good effect when you take VCO is that your skin is silk smooth, your face is fairer and if you take a shower, you don't need any lotion, because your skin is naturally oily. That is the positive effect. Your hair is also stronger*.

But Buli, a 29-year-old ex-drug user from Karawang, one of the most active members of the support group in Karawang says:

*The coconut oil, I was also suggested it, but I don't dare to try it, because I thought I'm taking ARV now, so it's better if I just continue*.

**Figure 3:**
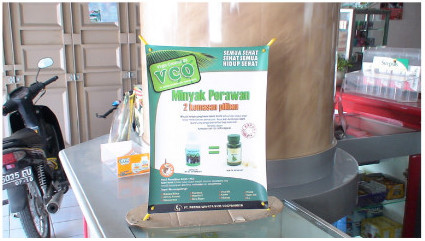
VCO, Indonesia.

In Indonesia, the drug sellers were not very willing to discuss the effects of VCO. They would acknowledge that indeed these drugs are used by PLWA, or they would deny knowing anything about the drugs. But their pharmacies are full of advertisements for the products and they have prominent positions on their shelves (see Figure 3).

Content analysis of the package information for VCO in Indonesia revealed that they are marketed as real 'cure-alls', i.e. to kill viruses and bacteria and/or strengthen the immune system, efficacy claims that we also found in West Africa. For example the package leaflet for Vicofit (manufactured by Sumber Dinamis in Bogor) states that the drug has "a high content of lauric acid which has anti-virus, anti-bacterials and anti-protozoa properties." And that it is "believed to help improve the health condition of those with cholesterol, diabetes, coronary heart disease, hepatitis C, HIV positive, cancer, prostate, uric acid, osteoporosis, influenza and weight problems". The package for Virjint (produced by PT Vermindo International) states that the medicine is safe for daily use and without side effects. It lists two dosages: one for prevention (2 × 2 capsules per day) and another for treatment (2 × 3 capsules per day). The leaflet stipulates that the indications are:

- "to increase energy and body stamina

- to increase body resistance (Meningkatkan daya tahan tubuh) against bacterial, viral and fungal pathogens

- to reduce weight

- anti-oxidant, anticancer, and anti-HIV

- to overcome uric acid, hypertension, stroke, heart disease, atherosclerosis, osteoporosis, influenza, hepatitis, chickenpox, herpes, TB, diabetes, epilepsy, eczema, liver, haemorrhoids, kidney, peradangan (burning sensation), infection, degenerative disease."

The packages cite clinical research conducted elsewhere (Philippines, USA) to give legitimacy to the products. For example the leaflet of Holistic Virgin Coconut Oil states: "Based on research conducted in the Philippines, Holistic Virgin Coconut Oil is very effective to fight against SARS and AIDS".

## Conclusion

One of the key characteristics of alternative medicines in Asia and Africa is that they move from one cultural and geographic space to another, apparently without being constrained by trade-barriers, or regulatory mechanisms. Some governments promote the production and diffusion of neo-traditional medicines. They do so for economic reasons: alternative medicines are big business, but they also do so for ideological reasons: neo-traditional medicines reflect an attractive hybrid of modernity and national heritage, providing a sense of national identity in the globalized health economy [10]. The governments of India, China, Indonesia, and some African countries support research programs to further advance these neo-traditional products, and facilitate market diffusion. While registered pharmaceuticals are regulated heavily upon market entry (proof of efficacy is assessed by national drug regulatory authorities), this is not the case for alternative medicines. ART programs, which are sponsored by the same governments, usually discourage the use of alternative medicines, fearing the toxicity of the drugs, or that these medicines will interact with anti-retroviral medication and lead to discontinuation of ART therapy [11]. Governmental agencies may have contradictory attitudes towards the use of alternative medicines for AIDS, discouraging it within ART programs and supporting it within divisions of traditional medicine. An exception is the Chinese government, which officially supports a complementary medicine program for AIDS care and research [12].

Mass-produced alternative medicines meet an increasing demand for health products, a trend which has been labelled the "commodification of health" [13,14]: from the slums of Djakarta to rural settings in Burkina Faso, people believe more and more that they need pharmaceutical 'things' to protect their health and to treat illness symptoms. People living with HIV and AIDS are particularly uncertain about their health and their future: ART may be accessible and improve health now, but they wonder if this will be the case in the future. This uncertainty makes them an attractive market for the 'best of both worlds', alternative medicines, which come with assertions of 'natural' safety and 'biomedical' efficacy [15]. However the case studies presented in Marseille suggest that people especially want to use alternative medicines to delay onset of ART, treat opportunistic infections, restore health and alleviate adverse effects once on ART. Immune-boosters are popular, though our case studies suggest that PLWA are often ambivalent about alternative medicines that claim anti-HIV efficacy.

The case studies make clear that the market of alternative medicines for HIV/AIDS is dynamic. It adapts to progress in biomedicine, which has produced potent anti-retroviral medications. In some cases, the efficacy claims for alternative medicines reinforce a biomedical paradigm for HIV/AIDS, and fit with a healthy living ideology promoted by AIDS care programs and support groups. More interdisciplinary research is needed on the experience of people living with HIV/AIDS with these alternative medicines, the ways in which the products and their representations move from one cultural setting to another, and on the ways in which these products interact (or not) with anti-retroviral therapy at pharmacological as well as psychosocial levels. More research is also needed to assess the economic impact of these therapies, since people seem to be spending much on these 'other' medicines while ART is provided for free. A blanket denial of the relevance of these products for the quality of life of PLWA does not make sense for patients, who need precise information that make clear which products are likely to have negative interactions with ART, and which ones could be beneficial. Unfortunately research on the interactions between alternative medicine and antiretroviral drugs is sparse [11]. To be able to inform patients better, more clinical research is needed on the benefits and risks of those alternative medicines that are perceived to be beneficial by people living with HIV and AIDS.

## Authors' contributions

The authors prepared papers for a joint seminar held in Aix-en-Provence, see reference list for the titles of the contributing papers. A summary of the insights from the papers was subsequently presented at the Marseille Impact Meeting, based on which a draft of this manuscript was written by AH and AD. All authors have contributed to the final manuscript.

## References

[B1] Kleinman A (1980). Patients and healers in the context of culture: an exploration of the borderland between anthropology, medicine and psychiatry.

[B2] Hardon A, Desclaux A (2007). Alternative and traditional treatments for AIDS in the time of ART in resource-poor settings: a comparative analysis of recent anthropological studies [Abstract 431]. Abstracts AIDS Impact 8th International Conference, Marseille.

[B3] Leslie C (1989). Indigenous pharmaceuticals, the capitalist world system, and Civilization.

[B4] Afdhal AF, Welsch RL, Van der Geest S, Whyte SR (1988). The rise of the modern jamu industry in Indonesia: a preliminary overview. The context of medicines in developing countries: studies in pharmaceutical anthropology.

[B5] Bode M (2004). Ayurvedic and unani health and beauty products: reworking India's medical traditions. PhD thesis.

[B6] Egrot M (2007). An overview on neotraditional medicines for HIV/AIDS in West Africa. NAARPS workshop Alternative and traditional treatments for HIV/AIDS, Aix en Provence, CReCSS.

[B7] Kyakuwa M (2007). Staying healthy while on HAART: the experiences of providers and patients on HAART in Uganda's resource limited settings. NAARPS workshop Alternative and traditional treatments for HIV/AIDS, Aix en Provence, CReCSS.

[B8] Simon E (2007). Non-conventional HIV/AIDS treatments in West-Africa: based on the case of Benin. Proceedings Naarps workshop Alternative and traditional treatments for HIV/AIDS, Aix en Provence, CReCSS.

[B9] Hardon A, Hidayana I, Imelda D (2007). On coconut oil, buah merah and other treatments used by people living with AIDS in west-Java. Proceedings Naarps workshop Alternative and traditional treatments for HIV/AIDS, Aix en Provence, CReCSS.

[B10] Micollier E (2007). Neo-traditional treatments for AIDS in China: national AIDS treatment policy and local/global use of TCM (Traditional Chinese Medicine) [Abstract 345]. Abstracts AIDS Impact 8th International Conference, Marseille.

[B11] Langlois-Klassen D, Kipp W, Jhangri GS, Rubaale T (2007). Use of traditional herbal medicine by AIDS patients in Kabarole District, western Uganda. Am J Trop Med Hyg.

[B12] Micollier E (2007). 'Facettes de la recherche médicale et de la gestion du VIH-sida dans le système de santé chinois: un autre exemple d'adaptation locale de la biomédecine' (An outline of AIDS medical research and management of HIV and AIDS in the Chinese public health system: another example of biomedicine localisation). Sciences Sociales et Santé (Social Sciences and Health).

[B13] Geest S Van der, Whyte S, Hardon A (1996). The anthropology of pharmaceuticals: a biographical approach. Annual review of anthropology.

[B14] Nichter M, Vuckovic N (1994). Agenda for an anthropology of pharmaceutical practice. Soc Sci Med.

[B15] Babb DA, Pemba L, Seatlanyane P, Charalambous S, Churchyard GJ, Grant AD (2007). Use of traditional medicine by HIV-infected individuals in South Africa in the era of antiretroviral theraphy. Psychol Health Med.

